# Gender differences in the association between adiposity and probable major depression: a cross-sectional study of 140,564 UK Biobank participants

**DOI:** 10.1186/1471-244X-14-153

**Published:** 2014-05-26

**Authors:** Zia Ul-Haq, Daniel J Smith, Barbara I Nicholl, Breda Cullen, Daniel Martin, Jason MR Gill, Jonathan Evans, Beverly Roberts, Ian J Deary, John Gallacher, Matthew Hotopf, Nick Craddock, Daniel F Mackay, Jill P Pell

**Affiliations:** 1 Institute of Health & Wellbeing, University of Glasgow, 1 Lilybank Gardens, Glasgow G12 8RZ, UK; 2 University of Edinburgh, Edinburgh, UK; 3 University of Cardiff, Cardiff, UK; 4 Kings College London, London, UK; 5 Institute of Public Health & Social Sciences, Khyber Medical University, Peshawar, Pakistan

**Keywords:** Adiposity, Obesity, Depression, Mental health, Mood disorder, UK Biobank

## Abstract

**Background:**

Previous studies on the association between adiposity and mood disorder have produced contradictory results, and few have used measurements other than body mass index (BMI). We examined the association between probable major depression and several measurements of adiposity: BMI, waist circumference (WC), waist-hip-ratio (WHR), and body fat percentage (BF%).

**Methods:**

We conducted a cross-sectional study using baseline data on the sub-group of UK Biobank participants who were assessed for mood disorder. Multivariate logistic regression models were used, adjusting for potential confounders including: demographic and life-style factors, comorbidity and psychotropic medication.

**Results:**

Of the 140,564 eligible participants, evidence of probable major depression was reported by 30,145 (21.5%). The fully adjusted odds ratios (OR) for obese participants were 1.16 (95% confidence interval (CI) 1.12, 1.20) using BMI, 1.15 (95% CI 1.11, 1.19) using WC, 1.09 (95% CI 1.05, 1.13) using WHR and 1.18 (95% CI 1.12, 1.25) using BF% (all p < 0.001). There was a significant interaction between adiposity and gender (p = 0.001). Overweight women were at increased risk of depression with a dose response relationship across the overweight (25.0-29.9 kg/m^2^), obese I (30.0-34.9 kg/m^2^), II (35.0-39.9 kg/m^2^) and III (≥40.0 kg/m^2^) categories; fully adjusted ORs 1.14, 1.20, 1.29 and 1.48, respectively (all p < 0.001). In contrast, only obese III men had significantly increased risk of depression (OR 1.29, 95% CI 1.08, 1.54, p = 0.006).

**Conclusion:**

Adiposity was associated with probable major depression, irrespective of the measurement used. The association was stronger in women than men. Physicians managing overweight and obese women should be alert to this increased risk.

## Background

Both depression and obesity are major public health problems. Worldwide, more than 350 million individuals suffer from depression [1]. As a contributor to the burden of morbidity, it is ranked third globally and first in middle and high income countries, with morbidity expected to rise further [2]. In the United Kingdom alone, around 1 in 20 adults experience an episode of depression annually, and it is the third most common reason by patients to consult their general practitioner [3]. Major depression carries a significant economic and health burden [4,5]. It is associated with increased physical comorbidity [6], reduced health-related quality of life (HRQoL) [7], and impaired function in work, school and family life [8], as well as increased mortality [9], including suicides [10]. The prevalence of obesity is increasing both in the UK and worldwide [11], leading to suggestions of an “obesity pandemic” or “globesity”. As with depression, we know that adiposity is associated with reduced physical well-being [12], poor HRQoL [13-15], and higher societal costs [16].

The relationship between these two important public health problems has been the focus of an increasing number of studies over recent years, but these studies have produced inconsistent results [17,18]. Some have reported positive associations [19] whilst others have reported negative [20] or no associations [21]. We previously showed that adiposity was significantly associated with poor overall HRQoL [13], but this was largely due to reductions in the physical component of HRQoL, with the mental component reduced only among morbidly obese and increased among overweight [14]. Furthermore, we found that poor mental health was confined to obese women under 45 years of age, and the apparent protective role of being overweight was confined to men aged 45–59 years [19].

A meta-analysis of population studies reported a pooled odds ratio (OR) of 1.26 (95% CI 1.17, 1.36) for the association between obesity and depression [22]. This association was only significant in women (OR 1.32). Of the 17 studies included, 16 used body mass index (BMI) as a measure of obesity. Another recent meta-analysis reported a pooled OR of 1.38 (95% CI 1.22, 1.57) for the association between central obesity and depression [23]. A total of 15 studies were included in this review, of which 14 used waist circumference (WC) as the measure of central obesity. Several studies showed that the association between obesity and depression is stronger in women [19,24]. In contrast, a recent large study demonstrated that adiposity was a significant predictor of depression only in men [25].

In these two recent meta-analyses, most used self-reported adiposity measurements, and many were not adjusted for important potential confounders such as socio-economic status, physical comorbidity, and use of psychotropic medications. Only BMI and WC have been used as measures of adiposity, and they were simply dichotomized into obese and not obese, thereby losing information on the relationship across the spectrum of adiposity such as whether there is a dose relationship. There is some evidence that the relationship between adiposity and depression varies according to the level of adiposity [26] and that waist-to-hip ratio (WHR) and body fat percentage (BF%) may also be associated with depression [27]. One recent, comparatively smaller German study (N = 4907) examined the association between obesity and depression, using the continuous measure of BMI, WC and WHR but not the direct measurement of BF% [28]. Overall, there is a paucity of larger studies which used other than BMI measures in exploring this association.

In this study we aimed to investigate the association between probable major depression and four different measurements of adiposity (BMI, WC, WHR and BF%), measured by trained staff using standard procedures and tools, across the whole range of adiposity (from underweight to class III obese). We also explored whether the associations varied by sex among a very large sample from the UK middle to old aged population, after adjusting for potential confounding factors, including medical comorbidity, use of psychotropic drugs, social deprivation and ethnicity.

## Methods

### Data source

We conducted a cross-sectional study using baseline data collected on UK Biobank participants. National Health Service (NHS), UK maintains the records of almost all individuals of the general population through general practitioners. Based on these records, about 5 million primary invitations were sent to the eligible individuals who were living within a reasonable travelling distance from the assessment centres (see Additional file 1: Figure S1). UK Biobank recruited 502,682 participants, aged 40–69 years, via 22 assessment centres across the United Kingdom between 2006 and 2010. The assessment of mood disorders was included only in the last two years, during which 172,751 participants were recruited [29].

### Data collection

Participants completed a series of computer based questionnaires followed by a face to face interview with trained research staff. The information collected included demographics (including sex, age, ethnicity, employment status, and postcode of residence), lifestyle factors (including smoking status and alcohol consumption), self-reported physician-diagnosed comorbidities (cardiovascular disease, hypertension, diabetes and cancer), current medication and past or current experience of depressive and manic symptoms.

Anthropometric measurements (including height, weight, WC, hip circumference and BF%) were measured by trained data collectors, using standard operating procedures. BF% was calculated using a Tanita BC-418MA body composition analyser. WHR was derived by dividing WC (measured by a Wessex non-stretchable sprung tape at the level of the umbilicus) by hip circumference (measured at the widest point using the same device). BMI was derived by dividing weight in kilograms (measured after removal of shoes and heavy outer clothing using a Tanita BC-418MA device) by the square of height in metres (measured without shoes using the Seca 202 device) [30].

### Definitions

BMI was classified as; underweight (<18.5 kg/m^2^), normal-weight (18.5-24.9 kg/m^2^), overweight (25.0-29.9 kg/m^2^), and obese (≥30 kg/m^2^). Obesity was further classified as class I (30.0-34.9 kg/m^2^), class II (35.0-39.9 kg/m^2^) or class III obese (≥40 kg/m^2^). Among men, WC was classified as normal-weight (<94 cm), overweight (94–101 cm), and obese (≥102 cm). The corresponding cut-off values for women were <80, 80–87 and ≥88 cm, respectively. WHR was classified among men as; normal weight (<0.90), overweight (0.90-0.99) or obese (≥1) and the corresponding cut-off values for women were <0.80, 0.80-0.84 and ≥0.85, respectively. BF% was classified among men as; normal weight (<18%), overweight (18-25%) and obese (>25%). The equivalent cut-off values for women were <25, 25–32 and >32, respectively.

Age was categorised into three groups; 39–49, 50–60, and 61–70 years. Townsend score quintile (from 1 least deprived to 5 most deprived) was used as an indicator of the participant’s socio-economic status. This is a validated measure which is determined on the basis of postcode of residence, and is derived from the following household information collected in the most recent census; car ownership, the number of people living in a house, home ownership, and employment status [31]. Frequency of alcohol consumption (daily/almost daily, 3–4 times/week, 1–2 times/week, 1–3 times/month, special occasions and never), smoking status (never, former and current), ethnic group (white, mixed, Asian/Asian British [Indian, Pakistani, Bangladeshi and other Asian background], black/black British, Chinese and other) and employment status (in paid employment, retired, looking after home, unemployed, not working due to sickness or disability and student) were self-reported. Comorbidity was defined as self-report of a doctor’s diagnosis of one or more of the following conditions; cardiovascular disease (coronary heart disease or stroke), hypertension, diabetes or cancer. Text information on all current medications was used to identify participants taking “any psychotropic medication” based on a list of 125 eligible generic and proprietary names compiled by three psychiatrists (DS, BC and DM).

Our classification of probable major depression was based on criteria published previously by our group [32]. We convened a series of meetings of Biobank-approved researchers focusing on mental health and cognition (membership DJS, JPP, DM, NC, JG, MH, BC, BN, DM, JE, ID and BR) and, after a number of iterations of proposed criteria, a definition for probable major depression was agreed. It should be noted that this approach represented a pragmatic synthesis of the data which was available to us as part of the UK Biobank baseline assessments and that the validity of this diagnosis is in part supported by differences between the probable depression group and controls in terms of gender distribution, socioeconomic status, self-reported health rating, current depressive symptoms and smoking status. Probable major depression and current depressive symptoms were therefore defined using information from specific questions on the severity and duration of both depressed mood and anhedonia, questions on past help-seeking behaviour for mental health and answers to the Patient Health Questionnaire (PHQ) [33]. Participants were then classified as having probable major depression if they reported a lifetime history of having ever had *either* depressed mood for a period of at least two weeks or a period of at least two weeks of being unenthusiastic/disinterested (anhedonic); plus they had reported ever having seen a general practitioner or psychiatrist for ‘nerves, anxiety, depression” in the past. We included participants who reported one or more eligible episodes but participants with probable bipolar I or II disorders were excluded from this study.

This study was conducted under generic approval from the National Health Service (NHS) National Research Ethics Service (17^th^ June 2011, Ref 11/NW/0382). Participants provided electronic consent for the baseline assessments, biochemical samples and future linkage to routine databases. Participants are not provided with individual level information nor benefit from any future commercial developments.

### Statistical analyses

The differences in depression and other covariates by adiposity were analysed using the *χ*^2^ test for categorical data, and *χ*^2^ test for trend for ordinal data. We examined the associations between anthropometric measurements (BMI, WC, WHR and BF%) and probably major depression, as the outcome, using multivariate logistic regression models. The association was first adjusted for age, sex, socio-economic status and ethnicity (model 1), and was then further adjusted for employment, alcohol consumption, smoking, presence of comorbidity (CVD, hypertension, diabetes, cancer) and use of psychotropic medications (model 2). We tested whether there were statistically significant interactions between adiposity and sex, and conducted sub-group analyses accordingly. The logistic regression model was repeated using BMI, WC, WHR and sex-specific deciles of BF%. All statistical analyses were performed using Stata version 12.1 (StataCorp, College Station, Texas). For the descriptive analysis the statistical significance was defined as p < 0.001.

## Results

Of the 172,751 UK Biobank participants who were recruited during the last two years, complete information on mood disorders was available for 140,564 (81.4%). Overall, the mean age was 57 years (SD 8 years), and 75,093 (53.4%) were women. 30,145 (21.5%) participants satisfied our criteria for probable major depression: 19,493 (26.0%) women and 10,652 (16.3%) men. Based on BMI, 33,857 (24.1%) were obese. Using the other measures, the percentage classified as obese were 46,504 (33.1%) for WC, 33,049 (23.5%) for WHR, and 91,166 (64.9%) for BF%. Depression was significantly more prevalent among women than men (19,493 [25.7%] versus 10,652 [16.3%], p < 0.001).

**Figure 1:**
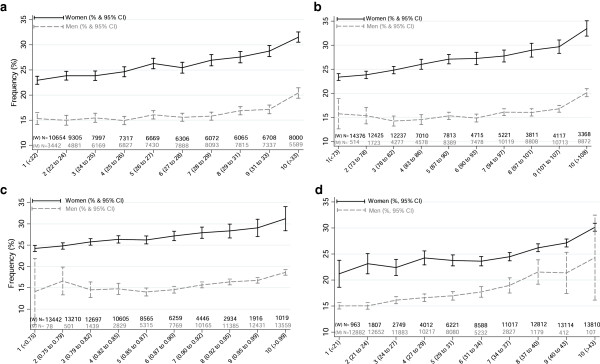
Frequency (%) of probable major depression by measures of adiposity and sex (a. Body Mass Index deciles; b. Waist Circumference deciles; c. Waist to hip ratio deciles; d. Body fat % deciles).

Those with probable major depression were more likely to be obese and were more likely to be women, younger, deprived, unemployed, white, smoke, report comorbidity, and use psychotropic medication, but they consumed alcohol less frequently (all p < 0.001) (Table 1). There was a positive association whereby probable major depression was less common in the lower deciles of adiposity and more common in the higher deciles, and this was more marked among women (Figure 1). In women, the prevalence of depression in the top decile of adiposity was very consistent across the different anthropometric measurements; 31.6%, 33.5%, 31.2% and 30.6% using BMI, WC, WHR, and BF% respectively. The corresponding proportions for men were 20.4%, 20.2%, 18.6% and 24.3%, respectively (Figure 1).

**Table 1:** Characteristics of the participants by body mass index category

				**Obese**				
	**Underweight**	**Normal-weight**	**Overweight**	**Overall**	**Class I**	**Class II**	**Class III**	**P-value**
	**N = 654**	**N = 46,121**	**N = 59,932**	**N = 33,857**	**N = 24,458**	**N = 6,852**	**N = 2,547**	
	**N (%)**	**N (%)**	**N (%)**	**N (%)**	**N (%)**	**N (%)**	**N (%)**	
**Probable major depression**								
No	508 (77.7)	36,622 (79.4)	47,641 (79.5)	25,648 (75.8)	18,912 (77.3)	5,022 (73.3)	1,714 (67.3)	<0.001
Yes	146 (22.3)	9,499 (20.6)	12,291 (20.5)	8,209 (24.3)	5,546 (22.7)	1,830 (26.7)	833 (32.7)	
**Sex**								
Women	517 (79.1)	29,748 (64.5)	27,380 (45.7)	17,448 (51.5)	11,634 (47.6)	4,089 (59.7)	1,725 (67.7)	<0.001
Men	137 (21.0)	16,373 (35.5)	32,552 (54.3)	16,409 (48.5)	12,824 (52.4)	2,763 (40.3)	822 (32.3)	
**Age (years)**								
39-49	170 (26.0)	12,200 (26.5)	12,958 (21.6)	7,119 (21.0)	4,970 (20.3)	1,478 (21.6)	671 (26.3)	<0.001
50-60	283 (43.3)	16,955 (36.8)	21,091 (35.2)	12,652 (37.4)	8,937 (36.5)	2,654 (38.7)	1,061 (41.7)	
61-70	201 (30.7)	16,966 (36.8)	25,883 (43.22)	14,086 (41.6)	10,551 (43.1)	2,720 (39.7)	815 (32.0)	
**Townsend score quintile**								
1 (least deprived)	107 (16.4)	8,289 (18.0)	10,552 (17.6)	4,795 (14.2)	3,718 (15.2)	818 (11.9)	259 (10.2)	<0.001
2	108 (16.5)	9,580 (20.8)	12,377 (20.7)	6,115 (18.1)	4,632 (18.9)	1,126 (16.4)	357 (14.0)	
3	111 (17.0)	9,563 (20.7)	12,795 (21.4)	6,734 (19.9)	4,956 (20.3)	1,314 (19.2)	464 (18.2)	
4	160 (24.5)	10,541 (22.9)	13,232 (22.1)	7,828 (23.1)	5,578 (22.8)	1,646 (24.0)	604 (23.7)	
5 (most deprived)	168 (25.7)	8,148 (17.7)	10,976 (18.3)	8,385 (24.8)	5,574 (22.8)	1,948 (28.4)	863 (33.9)	
**Employment status**								
In paid employment	339 (51.8)	27,597 (59.8)	34,155 (57.0)	18,733 (55.3)	13,651 (55.8)	3,704 (54.1)	1,378 (54.1)	<0.001
Retired	186 (28.4)	14,798 (32.1)	21,733 (36.3)	11,743 (34.7)	8,676 (35.5)	2,333 (34.1)	734 (28.8)	
Look after home	56 (8.6)	1,796 (3.9)	1,350 (2.3)	813 (2.4)	530 (2.2)	190 (2.8)	93 (3.7)	
Unemployed/unpaid	32 (4.9)	1,113 (2.4)	1,509 (2.5)	1,031 (3.1)	673 (2.8)	245 (3.6)	113 (4.4)	
Not working (sick/disable)	39 (6.0)	681 (1.5)	1,041 (1.7)	1,431 (4.2)	854 (3.5)	355 (5.2)	222 (8.7)	<0.001
Only student status	2 (0.3)	136 (0.3)	144 (0.2)	106 (0.3)	74 (0.3)	25 (0.4)	7 (0.3)	
**Alcohol consumption**								
Daily	139 (21.3)	10,604 (23.0)	13,137 (21.9)	5,337 (15.8)	4,296 (17.6)	846 (12.4)	195 (7.7)	<0.001
3-4 times/week	127 (19.4)	11,283 (24.5)	14,456 (24.1)	6,528 (19.3)	5,122 (20.9)	1,077 (15.7)	329 (12.9)	
1-2 times/week	123 (18.8)	11,419 (24.8)	15,411 (25.7)	8,677 (25.6)	6,398 (26.2)	1,720 (25.1)	559 (21.6)	
1-3 times/month	71 (10.9)	4,775 (10.4)	6,345 (10.6)	4,643 (13.7)	3,152 (12.9)	1,065 (15.5)	426 (16.7)	
Special occasions only	94 (14.4)	4,587 (10.0)	6,270 (10.5)	5,306 (15.7)	3,299 (13.5)	1,358 (19.8)	649 (25.5)	
Never	100 (15.3)	3,453 (7.5)	4,313 (7.2)	3,366 (9.9)	2,191 (9.0)	786 (11.5)	389 (15.3)	
**Smoking status**								
Never	387 (59.2)	27,887 (60.5)	32,729 (54.6)	17,652 (52.1)	12,614 (51.6)	3,652 (53.3)	1,386 (54.4)	<0.001
Previous	151 (23.1)	13,590 (29.5)	21,537 (35.9)	13,154 (38.9)	9,575 (39.2)	2,638 (38.5)	941 (37.0)	
Current	116 (17.7)	4,644 (10.1)	5,666 (9.5)	3,051 (9.0)	2,269 (9.3)	562 (8.2)	220 (8.6)	
**Ethnicity**								
White	591 (90.4)	42,911 (93.0)	55,312 (92.3)	30,838 (91.1)	22,382 (91.5)	6,191 (90.4)	2,265 (88.9)	<0.001
Mixed	10 (1.5)	377 (0.8)	381 (0.6)	235 (0.7)	161 (0.7)	47 (0.7)	27 (1.1)	
Asian/Asian British	28 (4.3)	1,426 (3.1)	1,893 (3.2)	863 (2.6)	653 (2.6)	162 (2.4)	48 (1.9)	
Black/Black British	3 (0.5)	651 (1.4)	1,521 (2.5)	1,451 (4.3)	926 (3.8)	355 (5.2)	170 (6.7)	
Chinese	6 (0.9)	291 (0.6)	169 (0.3)	38 (0.1)	34 (0.1)	3 (0.0)	1 (0.0)	
Other	16 (2.5)	465 (1.0)	656 (1.1)	432 (1.3)	302 (1.2)	94 (1.4)	36 (1.4)	
**Comorbidity**								
No	503 (76.9)	35,056 (76.0)	38,488 (64.2)	16,354 (48.3)	12,551 (51.3)	2,920 (42.6)	883 (32.7)	<0.001
Yes	151 (23.1)	11,065 (24.0)	21,444 (35.8)	17,503 (51.7)	11,907 (48.7)	3,932 (57.4)	1,664 (65.3)	
**Psychotropic medication**								
No	605 (92.5)	43,487 (94.3)	56,091 (93.6)	30,475 (90.0)	22,263 (91.0)	6,059 (88.4)	2,153 (84.5)	<0.001
Yes	49 (7.5)	2,634 (5.7)	3,841 (6.4)	3,382 (10.0)	2,195 (9.0)	793 (11.6)	394 (15.5)	

**BMI**: body mass index category (kg/m^2^); underweight (<18.5), normal-weight (18.5-24.9), overweight (25–29.9), obese (≥30), class I (30–34), class II (35–39), class III obese (>40), Townsend score, a measure of socio-economic status, p-value; χ^2^ test for categorical data & p-value for test of trend for ordinal data, Comorbidity (CVD, hypertension, diabetes, cancer).

In the overall logistic regression analyses, adjusted for age, sex, socio-economic status and ethnicity (model 1) there were significant associations between all anthropometric measures and probable major depression (all p-value <0.001) (Table 2). Being overweight or obese was significantly associated with probable major depression, and the odds ratio of major depression in obese participants was very consistent across the anthropometric measurements; 1.36, 1.34, 1.30, and 1.32 for BMI, WC, WHR and BF%, respectively (Table 2). When further adjusted for the potential confounding effects of employment, alcohol consumption, smoking, comorbidity (cardiovascular disease, hypertension, diabetes, and cancer) and use of psychotropic medications (model 2), the associations were slightly attenuated. Nonetheless, participants classified as overweight or obese (class I, II or III) based on BMI still had significantly higher odds of having probable major depression, compared to normal weight participants, with evidence of a linear relationship. The odds ratios of probable major depression were; 1.09, 1.12, 1.21, and 1.39 (all p-value <0.001) for overweight, class I, II and III obese, respectively. Similarly, using WC, WHR, and BF%, overweight and obese participants had significantly higher odds of probable major depression than normal-weight participants, with a dose–response relationship. The odds ratios for the association between overweight and probable major depression compared to normal weight remained very consistent across the anthropometric measurements; 1.09, 1.07, 1.05 and 1.06 for BMI, WC, WHR and BF% respectively. The corresponding odds ratios for obese participants were 1.16, 1.15, 1.09 and 1.18, respectively (Table 2).

**Table 2:** Logistic regression analysis of the adiposity measurements associated with probable major depression

		**Overall**				**Women**				**Men**			
		**Model 1**		**Model 2**		**Model 1**		**Model 2**		**Model 1**		**Model 2**	
**N = Overall (women, men)**		**OR (95% CI)**	**P-value**	**OR (95% CI)**	**P-value**	**OR (95% CI)**	**P-value**	**OR (95% CI)**	**P-value**	**OR (95% CI)**	**P-value**	**OR (95% CI)**	**P-value**
**BMI category**													
Underweight	654 (517, 137)	1.00 (0.83, 1.20)	0.972	0.93 (0.77, 1.13)	0.480	0.92 (0.74, 1.13)	0.428	0.89 (0.71, 1.10)	0.281	1.47 (0.98, 2.21)	0.061	1.25 (0.82, 1.93)	0.302
Normal-weight	46,121 (29748,16373)	1	-	1	-	1	-	1	-	1	-	1	-
Overweight	59,932 (27380, 32552)	1.15 (1.11, 1.18)	<0.001	1.09 (1.06, 1.13)	<0.001	1.20 (1.15, 1.24)	<0.001	1.14 (1.10, 1.19)	<0.001	1.07 (1.01, 1.12)	0.018	1.02 (0.97, 1.08)	0.480
Obese (overall)	33,857 (17448, 16409)	1.36 (1.32, 1.41)	<0.001	1.16 (1.12, 1.20)	<0.001	1.43 (1.37, 1.50)	<0.001	1.24 (1.19, 1.30)	<0.001	1.24 (1.17, 1.32)	<0.001	1.05 (0.99, 1.12)	0.134
Class I	24,458 (11634, 12824)	1.29 (1.24, 1.34)	<0.001	1.12 (1.08, 1.17)	<0.001	1.35 (1.29, 1.42)	<0.001	1.20 (1.14, 1.26)	<0.001	1.18 (1.11, 1.26)	<0.001	1.02 (0.96, 1.10)	0.489
Class II	6,852 (4089, 2763)	1.47 (1.38, 1.56)	<0.001	1.21 (1.14, 1.29)	<0.001	1.51 (1.41, 1.63)	<0.001	1.29 (1.20, 1.40)	<0.001	1.38 (1.25, 1.53)	<0.001	1.10 (0.99, 1.23)	0.081
Class III	2,547 (1725, 822)	1.82 (1.66, 1.98)	<0.001	1.39 (1.27, 1.53)	<0.001	1.84 (1.66, 2.04)	<0.001	1.48 (1.32, 1.65)	<0.001	1.76 (1.50, 2.08)	<0.001	1.29 (1.08, 1.54)	0.006
**WC category**													
Normal-weight	56,210 (29235, 26975)	1	-	1	-	1	-	1	-	1	-	1	-
Overweight	37,850 (18932, 18918)	1.13 (1.10, 1.17)	<0.001	1.07 (1.03, 1.10)	<0.001	1.15 (1.10, 1.20)	<0.001	1.09 (1.04, 1.14)	<0.001	1.11 (1.05, 1.17)	0.096	1.04 (0.98, 1.10)	0.161
Obese	46,504 (26926, 19578)	1.34 (1.30, 1.39)	<0.001	1.15 (1.11, 1.19)	<0.001	1.39 (1.33, 1.44)	<0.001	1.21 (1.16, 1.26)	<0.001	1.28 (1.22, 1.35)	<0.001	1.07 (1.02, 1.13)	0.010
**WHR category**													
Normal-weight	50,725 (31941, 18784)	1	-	1	-		-	1	-	1	-	1	-
Overweight	56,790 (19336, 37454)	1.14 (1.10, 1.18)	<0.001	1.05 (1.02, 1.09)	0.002	1.12 (1.08, 1.17)	<0.001	1.05 (1.01, 1.10)	0.024	1.18 (1.13, 1.24)	<0.001	1.06 (1.01, 1.12)	0.018
Obese	33,049 (23816, 9233)	1.30 (1.26, 1.35)	<0.001	1.09 (1.05, 1.13)	<0.001	1.26 (1.21, 1.31)	<0.001	1.09 (1.04, 1.13)	<0.001	1.46 (1.36, 1.56)	<0.001	1.12 (1.04, 1.20)	0.002
**BF% category**													
Normal-weight	10,186 (3829, 6357)	1	-	1	-	1	-	1	-	1	-	1	-
Overweight	39,212 (14774, 24438)	1.07 (1.01, 1.14)	0.016	1.06 (1.00, 1.13)	0.039	1.08 (1.00, 1.18)	0.061	1.10 (1.00, 1.20)	0.039	1.06 (0.98, 1.14)	0.156	1.03 (0.95, 1.12)	0.469
Obese	91,166 (56490, 34676)	1.32 (1.25, 1.40)	<0.001	1.18 (1.12, 1.25)	<0.001	1.37 (1.26, 1.48)	<0.001	1.26 (1.16, 1.37)	<0.001	1.27 (1.17, 1.36)	<0.001	1.09 (1.00, 1.18)	0.038

**OR**: Odds ratio; **CI**: Confidence Interval**; Model 1**: adjusted by age, sex, socio-economic status, and ethnicity; **Model 2 (**full adjusted): in addition to the variables in model-1 below variables were added: employment, alcohol consumption, smoking, comorbidity (CVD, diabetes, hypertension, cancer), and use of psychotropic medications; **BMI**: body mass index category (kg/m^2^); underweight (<18.5), normal-weight (18.5-24.9), overweight (25–29.9), obese overall (>30), class I (30–34), class II (35–39), and class III (>40); **WC**: Waist Circumference category (cm) men/women; normal-weight (<94/<80), overweight (94-101/80-87), obese (≥102/≥88); **WHR**: Waist-to-hip ratio category men/women; normal-weight (<0.90/<0.80), overweight (0.90-0.99/0.80-0.84), obese (≥1/≥0.85); **BF**: Body fat (%) men/women; normal-weight (<18/<25), overweight (18-25/25-32), obese (>25/>32).

There was a significant interaction between adiposity and sex (p = 0.001). Sub-group analyses by sex showed that the overall associations were largely driven by women (Table 2). In contrast, men classified as overweight, overall, class I or class II obese on the basis of their BMI were not at significantly increased risk of with probable major depression. Only class III obese men had significantly higher odds of probable major depression, compared to normal weight men. Similarly, using WC and BF%, there was no association between being overweight and probable major depression in men. Only obese men had significantly higher odds of probable major depression. In contrast, using WHR, both overweight and obese men were at significantly increased risk of probable major depression (Table 2). Underweight individuals were not at significantly increased risk of depression either overall or by gender-specific sub-group.

**Figure 2:**
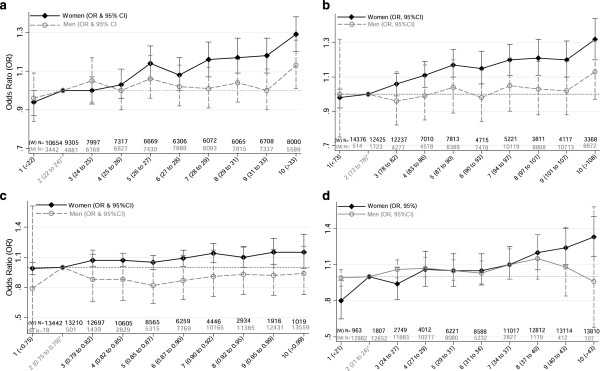
**Adjusted**^**¶ **^**odds ratio of probable major depression by measures of adiposity and sex (a. Body Mass Index deciles; b. Waist Circumference deciles; c. Waist to hip ratio deciles; d. Body fat % deciles).**

When the logistic regression model was repeated using the BMI, WC, WHR and BF% sex-specific deciles, the adjusted odds ratios in women illustrated the similar positive association (Figure 2) as was observed for the crude frequencies (Figure 1). The adjusted odds ratios for the top decile of BMI, WC, WHR and BF% were; 1.38, 1.35, 1.16 and 1.67, respectively. In contrast, among men, other than the top decile of BMI (>33 kg/m^2^), there was a straight line indicating no significant relationship with probable major depression in all anthropometric measurements.

## Discussion

Overall, both overweight and obese individuals were significantly more likely to have probable major depression than normal weight participants, irrespective of the anthropometric measurement used, and independent of potential confounding factors. There was evidence of a dose relationship with the risk of depression increasing with the level of adiposity, above normal weight. Being underweight was not associated with probable major depression. The relationship between adiposity and depression varied significantly by sex, such that the overall association was largely driven by women. In contrast, only men with class III obesity were at significantly increased risk of probable major depression.

In recent meta-analyses, increased BMI was associated with significantly reduced physical health-related quality of life [14,15]. In contrast, psychological health-related quality of life was higher among overweight individuals and reduced significantly in only class III obese. Two meta-analyses have reported a significant association between obesity and depression with pooled odds ratios of 1.38 for 1.26 for BMI [22] and WC [23]. Wiltink et al. examined the association between obesity and depression and demonstrated similar positive relationship, using the different measurements: BMI, WC and WHR [28]. In another study, Wyshak demonstrated a positive association between obesity, measured by BF%, and depression, relative to non-obese participants (adjusted OR 1.70, 95% CI 1.20, 2.39, p = 0.002) [27]. Our findings are consistent with those of Wiltink et al. [28], in that the magnitude of the association between obesity and depression was comparable using a number of different measurements: 1.36, 1.34, 1.30, and 1.32 for BMI, WC, WHR and BF%, respectively and there was an overall dose response relationship across the categories from overweight to obese III.

We found that the relationship between adiposity and depression was stronger in women than men. Previous studies have reported that overweight individuals have better mental health than normal-weight individuals [34], but some have found that this was confined to middle-aged men [19]. It is plausible that the association may be causal, and stronger in women. Adiposity can result in stigma, particularly in women, which is a known risk factor for depression [35]. Print and electronic media portrayals of thin women and larger, muscular men as ideals may lead to a lower acceptance of increased body weight among women [36]. It is also plausible that reverse causation may play a role. Depression may lead to both less physical activity and over-eating, contributing to obesity [37]. Physical attractiveness is known to be associated with depression, and depression may reduce an individual’s general interest in maintaining their appearance [38]. Depressed people are also reported to have more realism or even to underestimate their physical attractiveness [39]. In contrast, feeling attractive may be protective against depression. Depression is also known to be associated with neuro-endocrine abnormalities, such as hypercortisolaemia, which can contribute to obesity [40]. In this cross-sectional study, we were unable to establish temporal relationships and, therefore, could not determine temporal relationships.

Only a small number of previous studies have examined the association between level of adiposity and depression and, to our knowledge, this is the first study to explore the whole range of adiposity (from underweight to class III obesity), not rely on self-reported adiposity and use four different measurements of adiposity. Use of UK Biobank data enabled us to analyze a very large number of participants recruited from the general population, and to adjust for a wide range of potential confounders. The importance of adjusting for medical comorbidity, use of psychotropic medications, ethnicity and socio-economic status has been highlighted previously [22,23,28], but this has rarely been carried out.

### Limitations

Inclusion in our study was limited to the participants who provided complete information on mood. Approximately 20% of participants provided some but not all of the answers to questions about depression, anhedonia, duration of symptoms and previous help-seeking behaviour and could not be classified with confidence as having a lifetime history of probable major depression or not. These individuals were more likely to be of normal-weight, men, younger, in employment, had a history of more alcohol use, were more socially deprived, more likely to be non smokers and did not report medical comorbidity as often as those participants who did not provide complete information. We also acknowledge that our definition of probable major depression is a pragmatic approach based on the data which were available to us rather than a formal structured diagnosis. As such, it is possible that we may have missed out some participants with a lifetime history of major depression who have never sought treatment for it. Further, a proportion of people who seek treatment for “nerves or anxiety” may also have had low mood and anhedonia without meeting full diagnostic criteria for major depression. For these reasons, we have been careful to classify participants in this study with significant depressive features as ‘probable major depression’ rather than formally diagnosed ‘major depressive disorder’.

UK Biobank recruited middle and old aged individuals (aged 40 to 69 years) from the general population and so young people or very old people are underrepresented. Less than 10% of invited individuals were recruited into UK Biobank. UK Biobank is representative of the UK population in terms of demographics, but may not be representative in terms of other parameters. However, this does not, necessarily impact on the generalisability of the findings. Previously, we reported an association between being underweight and poor mental health, particularly in women [19]. The lack of an association with underweight in this study may reflect a lack of statistical power due to smaller numbers in this sub-group, or may be due to the previous study using the General Health Questionaire (GHQ-12) which is a short screening instrument rather than a detailed assessment of mental health.

## Conclusions

Overweight and obese women are significantly more likely to suffer from probable major depression, and the risk increases with increasing level of adiposity, even after adjusting for a range of potential confounders. Physicians managing overweight and obese women should be alert to this increased risk. Further research is required into whether the associations are causal, the direction of causality, and whether obesity interventions can reduce the risk of depression.

## Abbreviations

HRQoL: Health-related quality of life; OR: Odds ratios; CI: Confidence interval; BMI: Body mass index; WC: Waist circumference; WHR: Waist-hip-ratio; BF%: Body fat percentage; PHQ: Patient health questionnaire; NHS: National health service; GHQ-12: General health questionaire.

## Competing interests

The authors declare that they have no competing interests. Dr. Zia Ul-Haq is sponsored by the Higher Education Commission, Pakistan (Development of Khyber Medical University, Peshawar).

## Authors’ contributions

All authors contributed to conception and design. ZUH analysed the data, and supervised by DFM and JP. All authors agreed what analyses were required and interpreted the results. ZUH wrote the first draft. All authors revised the manuscript and approved the final version and takes full responsibility for the manuscript.

## Pre-publication history

The pre-publication history for this paper can be accessed here:

http://www.biomedcentral.com/1471-244X/14/153/prepub

## Supplementary Material

Additional file 1: Figure S1 Schematic of UK biobank invitation and appointment system. Click here for file
