# Supplementary figures and images for: Gender differences in the association between adiposity and probable major depression: a cross-sectional study of 140,564 UK Biobank participants
Source: BMC Psychiatry. 2014 May 26;14:153. doi: 10.1186/1471-244X-14-153 (PMC4050096; doi:10.1186/1471-244X-14-153)

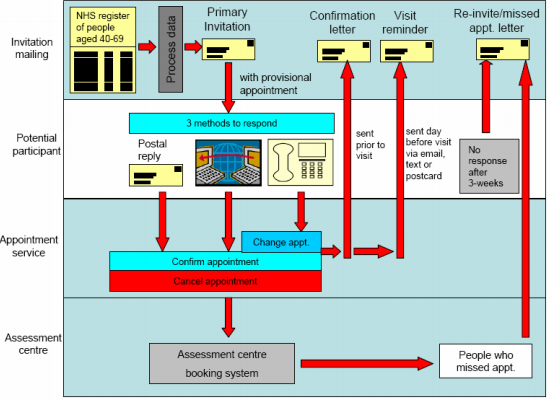

Supplement: Additional file 1: Figure S1 — Schematic of UK biobank invitation and appointment system. [file 1471-244X-14-153-S1.png]
